# Is fetal magnetic resonance imaging volumetry of eventrated organs in gastroschisis predictive for surgical treatment?

**DOI:** 10.1007/s00247-021-05066-z

**Published:** 2021-05-05

**Authors:** Patrick Sezen, Florian Prayer, Daniela Prayer, Gregor Kasprian, Martin Metzelder

**Affiliations:** 1 Department of Surgery, Division of Pediatric Surgery, Medical University of Vienna, Währinger Gürtel 18-20, 1090 Vienna, Austria; 2 Department of Biomedical Imaging and Image-guided Therapy, Medical University of Vienna, Vienna, Austria

**Keywords:** Fetus, Gastroschisis, Magnetic resonance imaging, Surgery, Volumetry

## Abstract

**Background:**

Fetal MRI is increasingly used in congenital abdominal wall defects. In gastroschisis, the role of fetal MRI in surgical therapy is poorly understood. Currently, the type of repair is determined primarily by clinical presentation and institutional preference.

**Objective:**

To evaluate the feasibility of fetal MRI volumetry in gastroschisis treatment.

**Materials and methods:**

We included 22 cases of gastroschisis in this retrospective single-center study. Routine fetal MRI scans were acquired between Jan. 1, 2006, and July 1, 2018, at gestational ages of 19–34 postmenstrual weeks. Fetal-MRI-based manual segmentation and volumetry were performed utilizing steady-state free precision and T2-weighted sequences. Acquired parameters included intraabdominal volume, eventrated organ volume and total fetal body volume, and we calculated a volume ratio between eventrated organ volume and intraabdominal volume (E/I ratio).

**Results:**

Primary closure was conducted in 13 cases and silo bag treatment with delayed closure in 9 cases. Prenatal MRI volumetry showed a significantly higher E/I ratio in patients with silo bag treatment with delayed closure (mean [M]=0.34; 95% confidence interval [CI] 0.30, 0.40) than in primary closure (M=0.23, 95% CI 0.19, 0.27; *P*=0.004). We propose a volume ratio cutoff value of 0.27 for predicting silo bag treatment.

**Conclusion:**

Fetal MRI predicted silo bag treatment in patients with gastroschisis in 90% of the cases in our cohort and might facilitate prenatal counseling and treatment planning.

**Supplementary Information:**

The online version contains supplementary material available at 10.1007/s00247-021-05066-z.

## Introduction

Gastroschisis describes a congenital abdominal wall defect allowing organ protrusion into the amniotic cavity. While the aetiology is poorly understood [1], theories regarding the development of gastroschisis involve vascular abnormalities [2], amniotic membrane rupture [3], teratogenic factors leading to abnormal mesenchymal differentiation [4] as well as failure of umbilical ring/umbilical cord attachment [5].

Gastroschisis treatment requires postnatal neonatal intensive care unit support with bowel protection, fluid management, respiratory support and thermoregulation [1]. Surgical treatment usually consists either of primary closure or initial silo bag placement with delayed closure. In a meta-analysis of randomized studies, silo bag treatment with delayed closure showed better outcome [6]. Further treatment options utilize sutureless closure using an umbilical cord flap [7], as well as patch closure using biomaterial, e.g., synthetic fluoropolymer patches [8, 9]. Immediate outcome in simple gastroschisis has been reported to be excellent. Survival rates exceed 90% and long-term quality of life is comparable to reference populations [1].

Routine antenatal sonographic screening in the 12th week of gestation has been shown to be highly sensitive regarding abdominal wall defect detection [10, 11]. Because of its widespread availability, sonography is regarded as the modality of choice for prenatal imaging of gastroschisis. Owing to excellent tissue contrast and wide field of view, fetal MRI is increasingly used in congenital malformations including abdominal pathologies [12]. Prognostic factors associated with poor outcome in gastroschisis are intestinal atresia, volvulus or perforation, which might be detected by prenatal imaging [13]. However, sonographic parameters like bowel wall thickness or maximum bowel dilatation have not shown consistent predictive value regarding outcome [14]. To date, similar parameters have not been investigated in fetal MRI, according to the literature. Recent studies regarding MRI in gastroschisis report morphological and descriptive characteristics for diagnosis and fetal development, but no standardized parameters regarding prediction of treatment or outcome [12, 13, 15, 16]. This study assessed fetal-MRI-based volumetry in fetuses with gastroschisis and evaluated its possible predictive value regarding surgical treatment.

## Materials and methods

The institutional review board of the Medical University of Vienna approved this retrospective study (Ethics Committee number 1493/2020).

We included all fetuses diagnosed with gastroschisis who underwent a fetal MRI scan and postnatal surgical treatment between Jan. 1, 2006, and July 1, 2018. Diagnosis of gastroschisis was established by routine obstetric sonography at the Department of Obstetrics and Gynecology. MR imaging was conducted at the Department of Biomedical Imaging and Image-guided Therapy. Routine fetal MRI was performed according to International Society of Ultrasound in Obstetrics and Gynecology (ISUOG) guidelines [17] and included steady-state free precision sequences in three orthogonal planes, and standard T2-W turbo spin-echo sequences (TSE), T1-W sequences acquired during maternal breathhold, and T2*-weighted sequences in coronal planes. Surgery was carried out at the Department of Surgery, Division of Pediatric Surgery. The pediatric surgeon decided the type of postnatal surgical treatment. Primary closure and silo bag treatment were conducted as described by Petrosyan and Sandler [18].

**Fig. 1:**
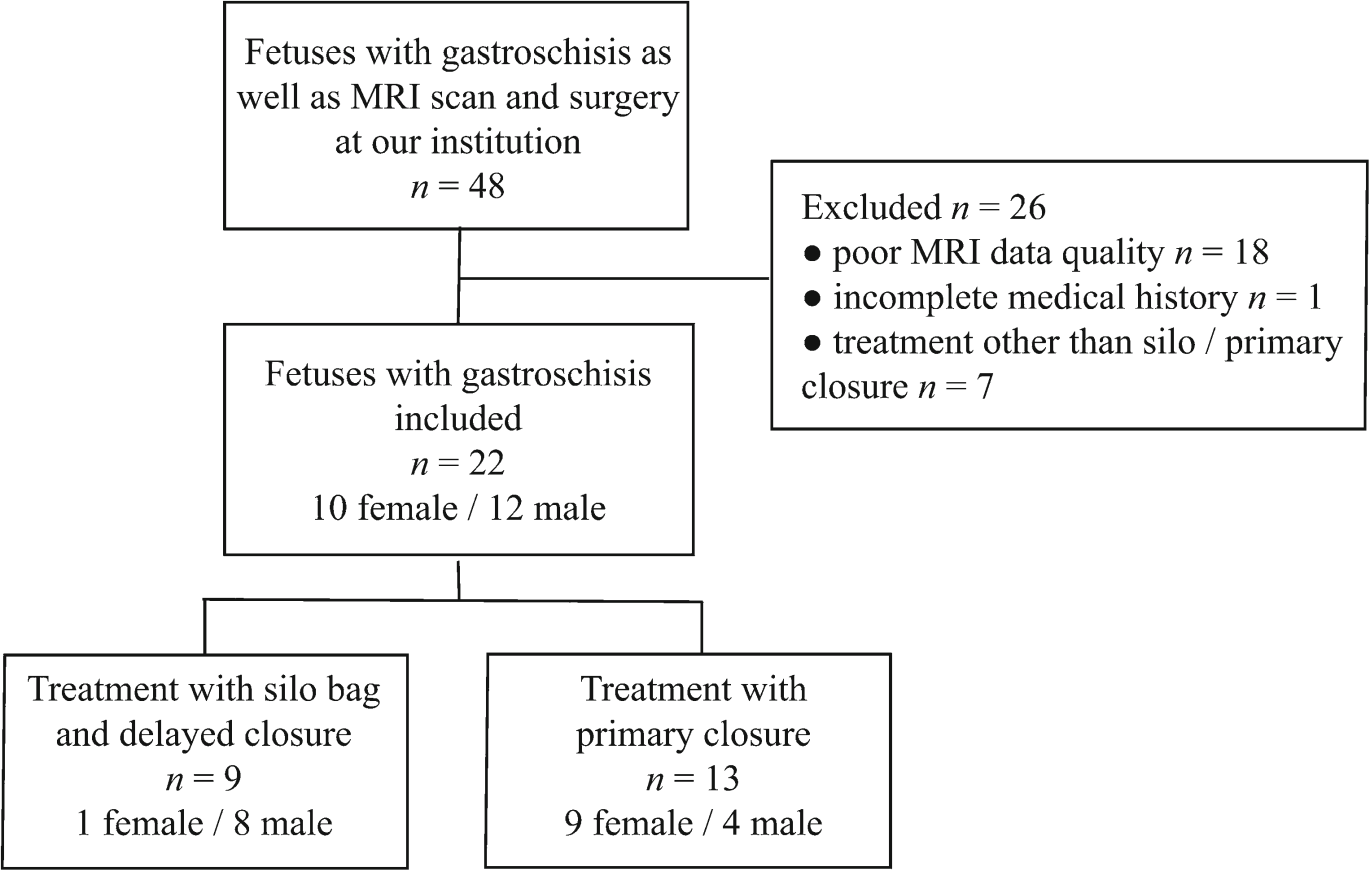
Flowchart shows the inclusion and exclusion criteria for the population of this study regarding fetal diagnosis of gastroschis undergoing MRI and surgery

A medical history review was conducted in 48 cases. Gestational age at time of scan was defined by prenatal sonography, gender by postnatal examination. We extracted MRI datasets from the institutional picture archiving and communication system and performed visual inspection of each image series. We excluded imaging with poor data quality regarding motion artifacts, wrap-around artifacts, completeness of fetus in a single imaging series, or lacking field of view or contrast between eventrated organs and umbilical vessels. Furthermore, we excluded cases with missing surgical reports or management that was different from silo bag treatment or primary closure. Based on these criteria, 22 of 48 subjects were included for volumetry and statistical analysis (Fig. 1).

### Fetal magnetic resonance imaging acquisition

Prenatal fetal MRI scans originated from several MR scanners: a 3-tesla (T) Achieva (Philips Medical Systems, Best, the Netherlands) with an eight-channel Sense cardiac coil, a 1.5-T Ingenia (Philips) with a 32-channel Multi Coil, or a 1.5-T Gyroscan Intera (Philips) with a five-channel Sense cardiac coil. The sequences used for volumetry were: for 1.5 T, either steady-state free precision (SSFP; slice thickness 6 mm, slice gap 2.5 mm, field of view [FOV] 256×256, matrix 192×219, repetition time [TR] shortest, echo time [TE] shortest, flip angle 80°) or T2-weighted turbo spin echo (TSE; slice thickness 4 mm, slice gap 3 mm, FOV 257×257, matrix 280×187, TR/TE 13,918/100 ms, flip angle 90°); and for 3 T, a T2-weighted TSE (slice thickness 2 mm, slice gap 2 mm, FOV 250×250, matrix 228×206, TR/TE 5,466/200 ms, flip angle 90°).

During the scan, the mother was in supine or left lateral position. No sedation or contrast agent was applied for the duration of image acquisition [19].

### Magnetic resonance imaging volumetry

**Fig. 2:**
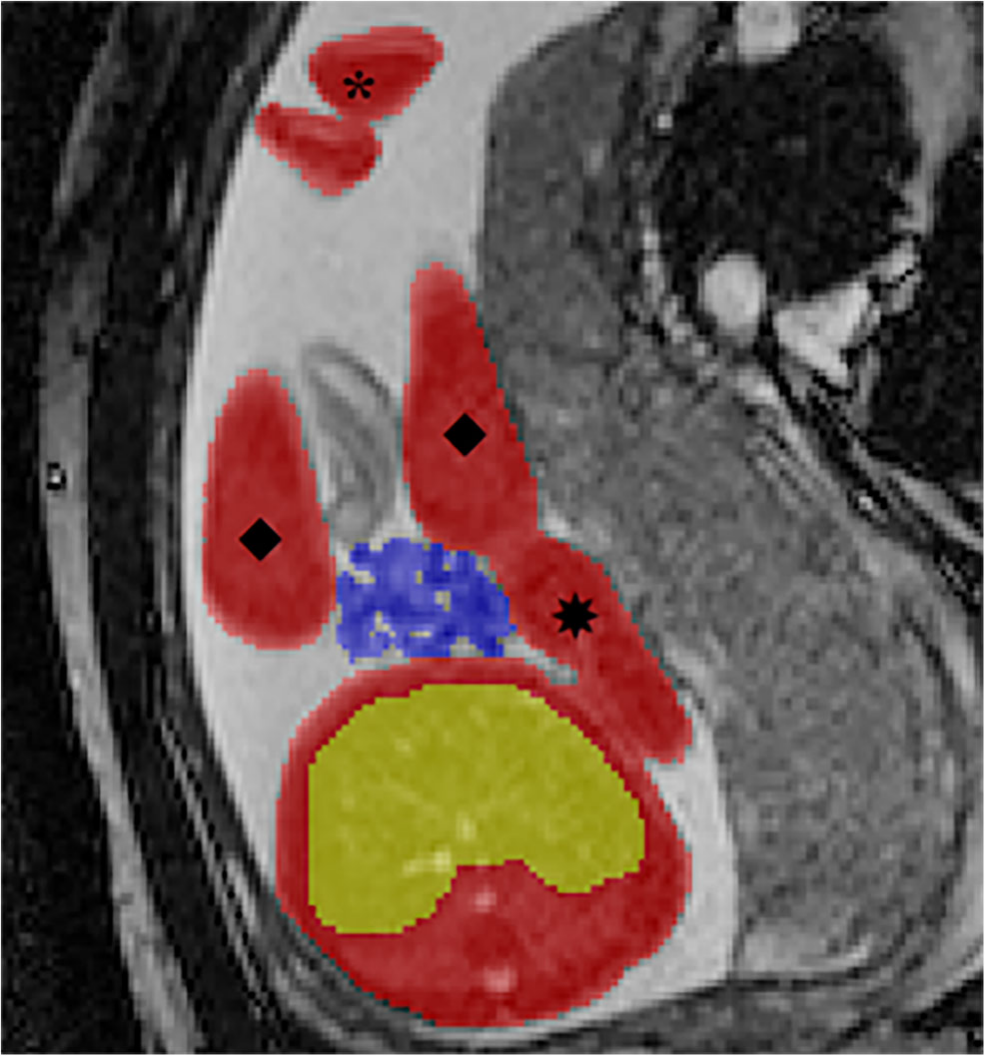
Manual segmentation in a fetus at 22 weeks of gestational age. Transverse view is shown in this slice of a steady-state free precession sequence. Eventrated intestine is seen in blue. It is flanked by upper (✴) and lower (◆) extremities. The unmapped part anterior is the umbilical vessels. The feet are distal from the rest of the body (*). In the central lower part of the picture is the torso, with the abdominal cavity in yellow and abdominal wall muscle, spine, back muscle and diaphragm/lung in red. On the right side of the picture is the placenta, next to the fetus

**Fig. 3:**
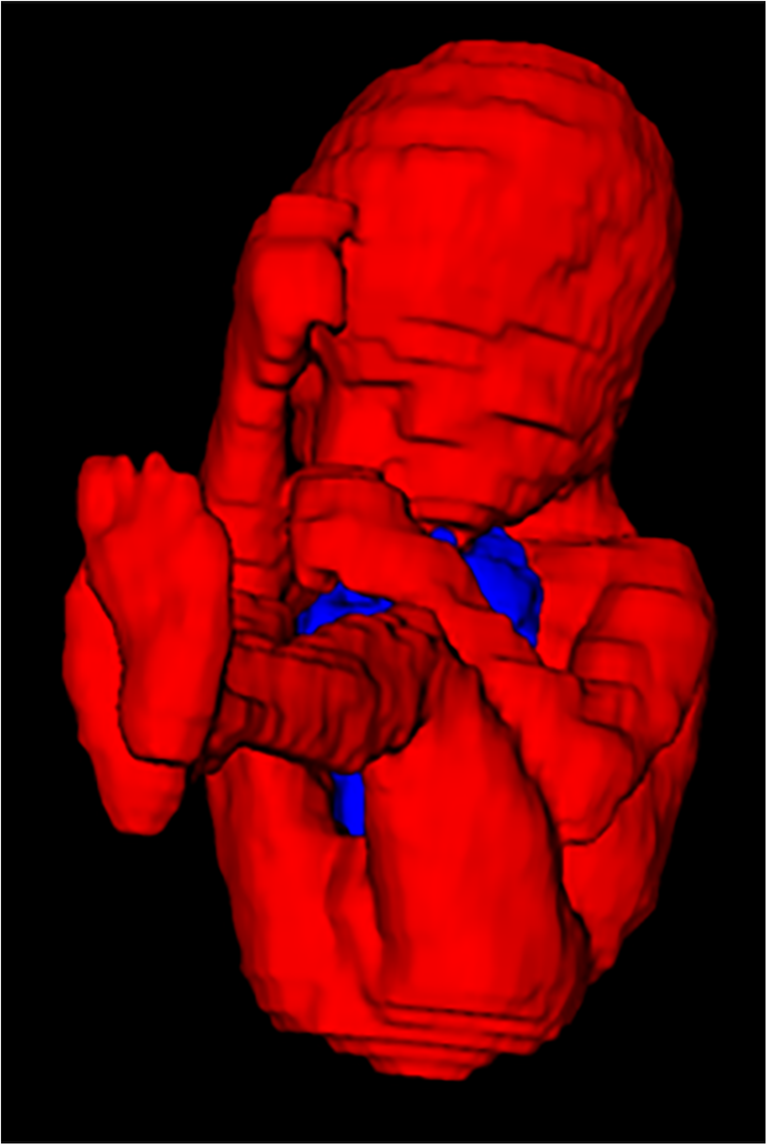
A three-dimensional overview of the same fetus as in Fig. 2 after full volumetry. The fetus is seen in red with the eventrated bowel between extremities and head seen in blue. Intraabdominal volume cannot be seen on this graphic

When possible, we chose SSFP sequences over T2-W sequences because of their superior umbilical vessel/eventration contrast. Volumetry was performed on a single plane, preferentially on transverse over sagittal orientation. We conducted the manual segmentation of eventrated organ volume, intraabdominal volume and total fetal volume using the open-source software application ITK-SNAP version 3.6.0 [20] (Fig. 2). Eventrated organ volume was identified and mapped first. To ascertain that umbilical vessels were not falsely labeled as eventrated intestine, we visually tracked the umbilical vessels from the placenta to the abdominal insertion. We determined the transition from eventrated organ volume to intraabdominal volume to be the plane where ventral abdominal muscle would have closed the abdominal cavity. Intraabdominal volume was mapped with the following anatomical borders. The superior boundary was formed by the thoracic cavity, respectively the diaphragm; delineation of intraabdominal organs from lung and heart was excellent, which in turn provided accurate mapping. The inferior border was defined at the height of the urinary bladder, which was not included in the intraabdominal volume; the kidneys were included in the intraabdominal volume. Anterior and lateral limits were the muscles of the abdominal wall. The posterior border was defined as the quadratus lumborum and psoas muscles as well as the spine, none of which was included in the intraabdominal volume. Figure 3 shows an example of the full volume in three dimensions. Full segmentation took approximately 100 min per subject on average. Segmentation of the intraabdominal and eventrated organ volume took about 20 min or less in most cases, while segmentation of total fetal volume took about 80 min.

After full segmentation, we generated volume in millimeters cubed (mm^3^) for each metric. The segmentation in this study was carried out by a single examiner (P.S.) in all cases. Because some degree of interrater variability is present in manual segmentation, a random sample of 10 cases was also examined by a second examiner (F.P.) with the same criteria as stated, in accordance with methodology previously used [21]. Both P.S. and F.P. were trained and overseen in volumetry and fetal MR imaging by senior radiologists with more than 15 years of experience in fetal MRI.

The generated volumes are eventrated organ volume and intraabdominal volume as measured directly. To calculate total fetal volume, we summed the eventrated organ volume, intraabdominal volume and the volume of the rest of the body.

### Statistical analysis

For statistical calculations, we used the RStudio IDE (RStudio Team 2016, version 1.1.456 desktop; RStudio, Boston, MA) [22], utilizing the free statistical software environment R (R Core Team 2018, version 3.5.1 Feather Spray; The R Project, Vienna, Austria) [23]. Plots were drawn using the package ggplot2 (version 3.0.0) [24]. Descriptive statistics include mean and confidence interval (CI) for gestational age at time of scan. Evaluation of group differences regarding gender, comorbidities, complications, gestational age at time of scan and gestational age at time of delivery was carried out with the Fisher exact test or Welch two-sample *t*-test. We tested group difference regarding subject count with chi-square test. To examine interrater variability, we used an intraclass correlation coefficient (ICC) calculated by a two-way, absolute agreement, random-effects model. ICC values of >0.9, <0.9 to 0.75, <0.75 to 0.5, and <0.5 were respectively regarded as excellent, good, moderate or poor agreement between raters.

A quotient between eventrated organ volume and intraabdominal volume was calculated (volume ratio). Differences in eventrated organ volume, intraabdominal volume and volume ratio between groups were evaluated by means of the Welch two-sample *t*-test. Considering effects of gestational age and total fetal volume on eventrated organs volume and intraabdominal volume, we used Pearson correlation coefficient to examine these relationships. Initial hypotheses regarding bias from heterogeneous gestational age or individual growth restrictions in our study were addressed by formulating a multiple linear regression model with the volume ratio as dependent variable and gestational age, total fetal volume, gender and treatment group as independent variables. The maximum Youden index was determined for the receiver operating characteristic (ROC) curve of volume ratio to determine the optimal cutoff point. We generated area under the curve (AUC) and confidence interval for AUC by bootstrapping. The two-sided significance level was *P*<0.05.

## Results

We included 22 subjects for analysis. Table 1 lists characteristics of the study population, allocation between treatment groups and respective results of statistical tests. The group treated with silo bag featured significantly more male subjects and less female subjects than the group treated with primary closure. Gestational age, comorbidities and complications across groups showed no significant differences. Only in the silo treatment group, there was a single case of complex gastroschisis with a small-bowel stenosis. Polyhydramnios was not present in any scan.

**Table 1:** Clinical characteristics

	**Silo bag**	**Primary closure**	**t**	**dF**	***P*-value**
Gestational age at MRI^a^, in weeks	25.1 (21.5, 28.7)	23.6 (21.6, 25.5)	0.86	12.91	0.41
Gestational age at delivery^a^, in weeks	36.5 (35.6, 37.4)	36.8 (36.2, 37.3)	−0.61	14.15	0.55
	**Silo bag**	**Primary closure**	**X**^**2**^	**dF**	***P*-value**
Number^b^	9	13	0.73	1.00	0.39
	**Silo bag**	**Primary closure**	**OR**	**95% CI**	***P*-value**
Gender^c^	M/F: 8/1	M/F: 4/9	0.065	0.001, 0.741	0.01
Comorbidities^c^	1	0	0.000	0.000, 27.00	0.41
Complications^c^	1	2	1.431	0.064, 96.32	1.00

*CI* confidence interval, *dF* degrees of freedom, *F* female, *M* male, *OR* odds ratio, *t* t-statistic, *X*^2^ chi-square value

^a^For both the gestational age at MRI and at delivery the mean is displayed with 95% CI in parentheses. A Welch *t*-test was conducted to check for group differences

^b^ Chi-square test for subject count (*n*) was conducted

^c^ Fisher exact tests were conducted for gender, comorbidities and complications

For intraabdominal volume, interrater agreement was excellent (ICC=.987; 95% CI 0.927, 0.997). Interrater agreement for eventrated organ volume was good (ICC=.885; 95% CI 0.63, 0.97).

Eventrated organ volume showed a significant correlation with both gestational age (r(20)=0.85, *P*<0.001) and total fetal volume (r(20)=0.92, *P*<0.001). Similarly, intraabdominal volume correlated significantly with both gestational age (r(20)=0.96, *P*<0.001) and total fetal volume (r(20)=0.99, *P*<0.001).

Eventrated organ volume (t(9.88)=1.64, *P*=0.13), intraabdominal volume (t(10.55)=1.19, *P*=0.26) and total fetal volume (t(10.85)=1.27, *P*=0.23) showed no significant differences between groups.

**Fig. 4:**
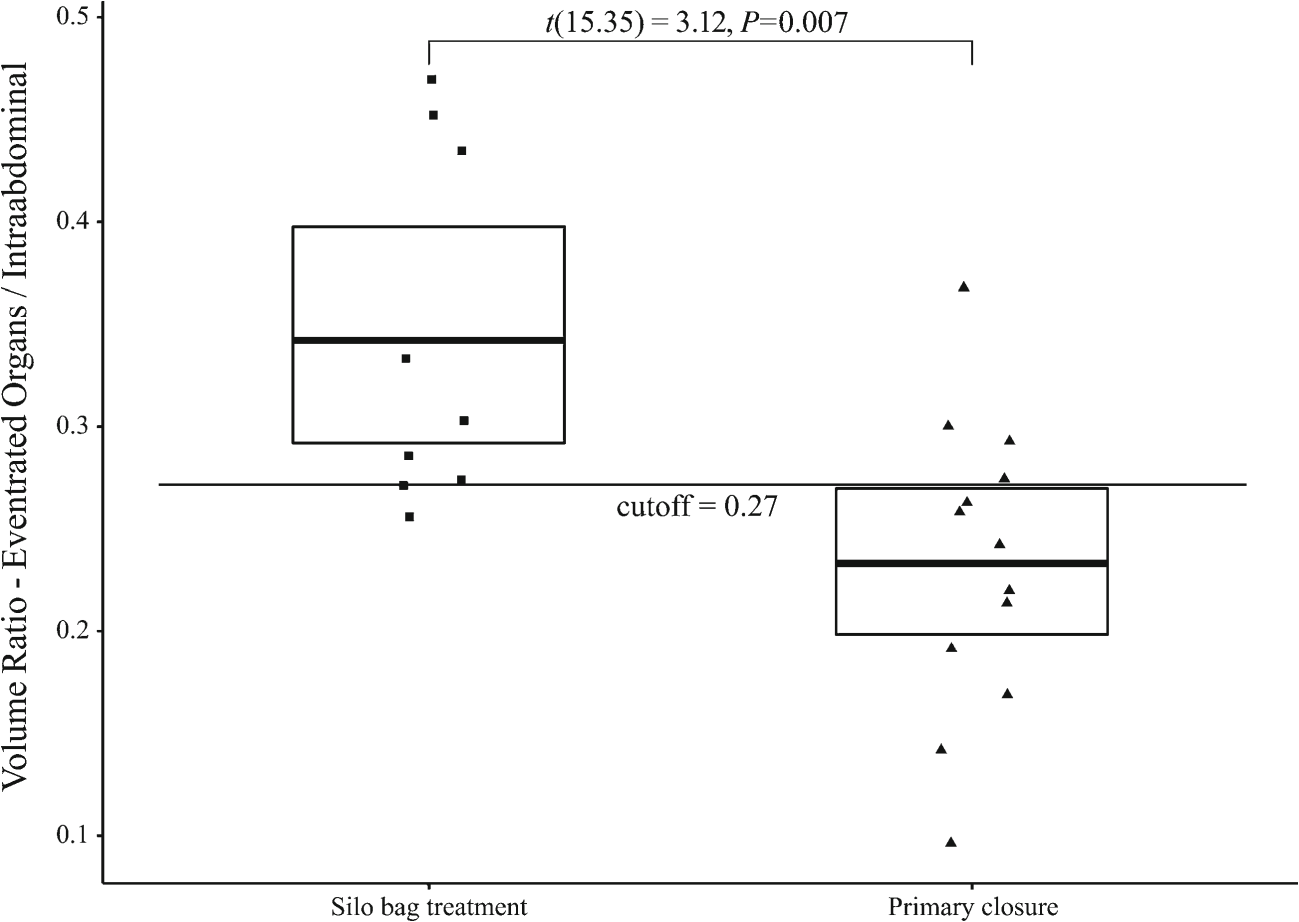
In this scatter boxplot, the ratio of eventrated organ volume and intraabdominal volume is shown for both treatment groups. Individual datapoints are depicted with a random jitter on the x-axis. Solid lines indicate mean values, while boxes represent a 95% confidence interval generated by bootstrapping. The proposed cutoff value for the volume ratio is shown as a single horizontal line at the volume ratio value of 0.27. A Welch *t*-test was conducted between groups. *t* t-statistic

A group-wise comparison between the ratio of eventrated organ volume and intraabdominal volume is presented in Fig. 4. Volume ratio in silo bag treatment group (mean [M]=0.34, 95% CI 0.30, 0.40) was significantly higher than in primary closure treatment group (M=0.23, 95% CI 0.19, 0.27). A version of this plot with separation of treatment groups by gender is in the online resource.

The results of the previously described multiple linear regression model to examine possible bias on volume ratio due to confounding variables is reported in Table 2. Treatment group as independent variable predicted volume ratio significantly. The other independent variables — gestational age, gender and total fetal volume — showed no significant explanatory effect on the observed variance of the volume ratio. As indicated by the R^2^, this model does not significantly explain variance in volume ratio. If independent variables other than treatment group are removed, adjusted R^2^ does increase, though, further indicating that gestational age, gender and total fetal volume do not explain variance in volume ratio sufficiently (F(1, 20)=10.38, *P*=0.004, R^2^=0.34, R^2^adjusted=0.31).

**Table 2:** Influence and significance of independent variables on estimated volume ratio^a^

	Coefficient	Standard error	t	*P*-value
Treatment group	−0.62	0.05	−2.55	0.02
Gestational age	−0.55	0.02	−0.63	0.54
Gender	−0.22	0.05	−0.86	0.40
Total fetal volume	0.68	0.00	0.75	0.46

*t* t-statistic

^a^Results of a multiple linear regression model with volume ratio between volume of eventrated organs and intraabdominal volume as the dependent variable, and treatment group, gestational age, gender and total fetal volume as independent variables

**Fig. 5:**
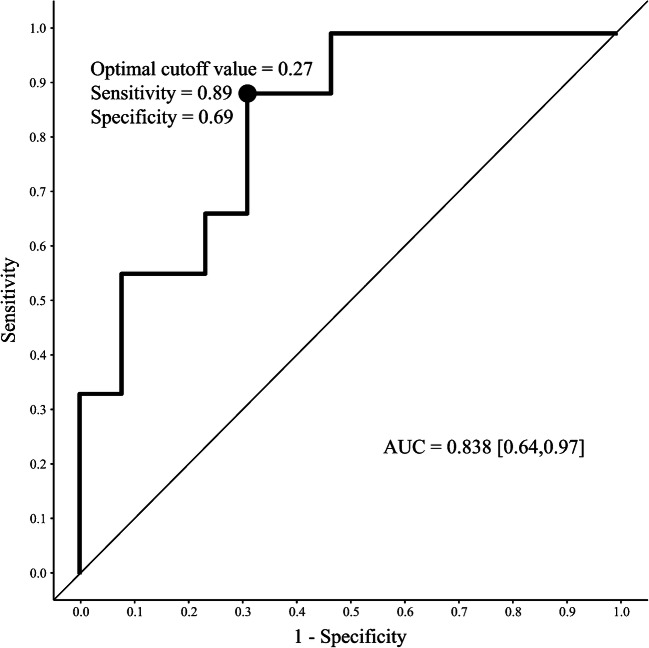
Receiver operating characteristic curve for volume ratio. Optimal cutoff value for volume ratio was determined by maximum of the Youden index. This cutoff value is also marked in Fig. 4. *AUC* area under the curve

An ROC curve was plotted for volume ratio as predictor for treatment group (Fig. 5). The AUC of 0.838 suggests a good ability of volume ratio to predict the treatment group. Respective positive predictive value for the optimal cutoff point was 0.67, while negative predictive value was 0.9.

## Discussion

In this retrospective cohort study, we demonstrated the feasibility of fetal-MRI-based volumetry in gastroschisis for the first time. There were good and very good interrater variabilities for manually mapping the eventrated organs, the intraabdominal space and the complete fetus.

We evaluated group differences between patients who received different kinds of postnatal treatment. Interestingly, patients who were treated initially with a silo bag and received consecutive reduction of eventration, with eventual delayed defect closure, showed an increased ratio of eventrated organ volume to intraabdominal volume in their prenatal fetal MRI scans. This suggests that silo bag treatment is more likely in patients with a higher volume ratio and could help predict and plan treatment weeks before planned delivery of the baby. We also proposed a cutoff value to guide counseling at different values of volume ratio. Regarding a causal relationship, it could be speculated that a higher portion of eventrated organs in conjunction with less intraabdominal space does not permit complete retrocession and closure of the abdominal wall, mainly because of abdominal compartment syndrome. Despite subjective surgical judgment and experience playing a role regarding management, more personalized imaging data should help support the decision process and preparedness of the treating pediatric surgeon.

Interestingly, in contrast to the significant differences in volume ratio, eventrated organ volume and intraabdominal volume without ratio were not significantly different between groups. This can be seen as evidence for both factors together leading to necessity for delayed closure and an initial period of dilating the abdominal cavity while compressing intestine through gravity from silo bag treatment.

Initially we assumed that gender differences between groups could induce bias because of reported growth differences in male and female fetuses [25] because our study population features uneven distribution regarding gender among treatment groups. But for the observed variability of volume ratio and its treatment group differences, it did not prove to have a significant influence. We also observed highly significant and strong correlations between the intraabdominal volume and volume of eventration, and the total fetal volume and gestational age in our sample. This finding seems intuitive because both could be seen as surrogate markers for growth in this regard, with gestational age having more of a systematic character and measured total fetal volume being more individual. As proved by linear regression, though, after calculating a volume ratio there was no significant effect of both age and total volume anymore.

The findings in our study have implications that could change current practice in prenatal counseling, as well as give surgeons a rough guide of what to expect treatment-wise in an individual patient. Prenatal MRI can be used not only to strengthen initial sonographic diagnosis and assess for additional comorbidities, it can also predict a treatment modality. With the proposed cutoff value for the volume ratio in this study, about 90% of subjects could be correctly predicted to receive silo bag treatment. On the other hand, about 67% of subjects could be correctly predicted to receive primary closure. We believe that with a bigger sample size this could be further improved. In turn, this would enable us to give more accurate prenatal counseling to parents, which could reduce fear and psychological stress. On the background that parents today are more well informed, they value a more precise diagnostic and therapeutic process. It would also help surgeons to plan the management, makes logistics around management more predictable, and could even serve as an objective diagnostic marker that could be taken into consideration when deciding upon whether primary closure or silo bag treatment and delayed closure is best for the small patients we treat.

### Limitations

Small sample size and therefore limited statistical power are known limitations of this study. A contributing factor in this regard is lacking MRI data quality, which led to initial exclusion of about half the data sets. As a consequence, image acquirement protocols have been changed to guarantee better image quality. Furthermore, with gastroschisis being a rare condition, sample size at a single institution will always be limited. The number of scans was further impeded by perceived limited indications for fetal MRI, according to a survey from 2014 [17].

Uneven distribution of gender among the treatment groups was a further limiting factor for this study. We made an effort to give a transparent picture regarding our data and we feel confident that gender does not affect the volume ratio.

To achieve a number of sufficient data sets, data collection had to be conducted over a timespan of 12 years, leading to additional problems regarding MRI data quality. Generated data were obtained with different MRI scanners and different protocols, which further limited homogeneity and potentially added bias to volumetric segmentation. Apart from that, resolution is limited in fetal MRI and movement artifacts are generally not controllable in absence of sedation, as opposed to MRI in infants in which non-pharmacological techniques like wrap-and-feed can be used [26].

Comorbidities, although scarce in our sample, also could lead to bias. One of the subjects included in this study did feature small-intestine stenosis in the terminal ileum, changing the diagnosis to complex gastroschisis postpartum. MRI data in that particular case were checked vigorously, and no dilation was observable. Nonetheless, signs of intestinal dilation or collapse from stenosis or atresia as well as other fetal malformations should be kept in mind as an indication for complex gastroschisis.

Given the retrospective nature of this study, we did not aim for outcome prediction. Measured volumes could not be compared to those of healthy subjects because of the absence of a healthy control group.

### Future perspective

Confirmation of these findings in a larger sample is necessary. Furthermore, manual segmentation is time-consuming, and bias from different raters is always a possibility. Approximation of volume by formula as done in other fields of imaging [27] or machine learning could offer the means to faster and more standardized volumetry [28].

## Conclusion

Our results suggest that fetal MRI volumetry can predict the need for silo bag treatment in gastroschisis with reasonable accuracy. Fetal MRI volumetry could better inform prenatal counseling and better prepare surgeons.

## Supplementary Information

ESM 1 (PDF 126 kb)
