# Supplementary material for: Is fetal magnetic resonance imaging volumetry of eventrated organs in gastroschisis predictive for surgical treatment?
Source: Pediatr Radiol. 2021 May 5;51(10):1818–25. doi: 10.1007/s00247-021-05066-z (PMC8426252; doi:10.1007/s00247-021-05066-z)
Supplement: Supplementary file 1 — (PDF 126 kb) [file 247_2021_5066_MOESM1_ESM.pdf]

Supplement

Title: Is fetal MRI-based volumetry of eventrated organs in gastroschisis predictive for surgical treatment? – A retrospective study

Journal: Pediatric Radiology

Authors: Patrick Sezen, Florian Prayer, Daniela Prayer, Gregor Kasprian, Martin Metzelder

Corresponding author:

Univ.-Prof. Dr. Martin Metzelder, FEAPU
Department of Surgery
Division of Pediatric Surgery
Medical University of Vienna
Währinger Gürtel 18-20
1090 Vienna, Austria

E-mail: martin.metzelder@meduniwien.ac.at

Fig. S1

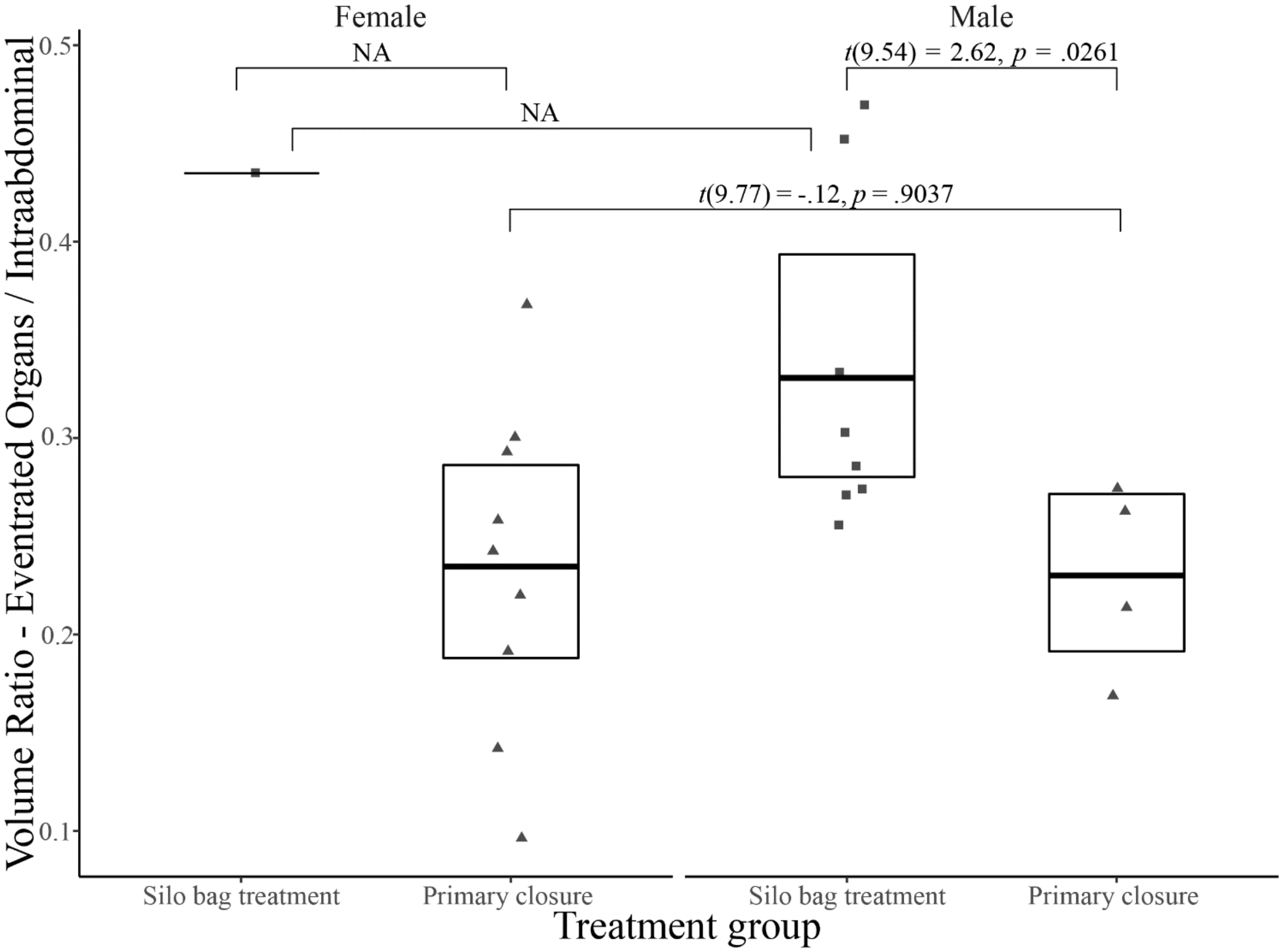

In this supplementary figure, which is analogous to Fig. 4 in the main article, gender subgrouping has been utilized. This should further demonstrate that there is no additional effect due to gender on treatment group, besides lacking subject count in the subgroup of female patients which received treatment with silo bag. Welch's t-test, which was used in this plot, is not possible to conduct for comparisons with a single subject in one group. Student's t-test can be used instead, although interpretation should be cautious: For the comparison between silo bag treatment group and primary closure group only in female subjects, no significant difference exists ($t(8) = 2.2739$, $p = .053$). Testing between female and male patients which were both treated with silo bag also showed no significant difference ($t(7) = 1.1722$, $p = .279$).
